# Spontaneous exudative retinal detachment in a patient with sturge-weber syndrome after taking arginine, a supplement for erectile dysfunction

**DOI:** 10.1186/s40662-014-0007-x

**Published:** 2014-10-22

**Authors:** Austin Bach, Aaron S Gold, Victor M Villegas, Andrea C Wildner, Fiona J Ehlies, Timothy G Murray

**Affiliations:** Larkin Community Hospital, 7031 SW 62nd Avenue, South Miami, 33143 Florida USA; Murray Ocular Oncology & Retina, 6705 Red Road, Suite 412, Miami, 33143 Florida USA

**Keywords:** Sturge-Weber Syndrome, Exudative retinal detachment, Choroidal hemangioma, Nitric oxide, arginine

## Abstract

**Background:**

Patients with Sturge-Weber syndrome can have ipsilateral diffuse or circumscribed choroidal hemangiomas. These hemangiomas have been seen to undergo spontaneous exudative or hemorrhagic retinal detachments. There is no definitive treatment for these types of retinal detachments, but radiotherapy, photodynamic therapy, oral propranolol, pegaptinib and bevacizumab have been used.

**Case presentation:**

A 26-year-old male with Sturge-Weber Syndrome developed an exudative retinal detachment that occurred immediately after taking a supplement containing arginine. The patient was treated with intravitreal bevacizumab 1.25 mg in 0.05 ml solution. Resolution of the retinal detachment was seen after 4 treatments over a six-month period.

**Conclusions:**

Arginine and other medications that cause a release of nitric oxide may lead to intravascular leakage and exudative retinal detachments in patients who have a choroidal hemangioma.

**Electronic supplementary material:**

The online version of this article (doi:10.1186/s40662-014-0007-x) contains supplementary material, which is available to authorized users.

## Background

Sturge-Weber Syndrome is a relatively uncommon neuro-oculo-cutaneous syndrome. The most noticeable feature is the nevus flammeus (aka port wine stain) involving dermatomal distribution of any one or multiple divisions of the trigeminal nerve. Diffuse or circumscribed choroidal hemangiomas are a common ocular feature of this syndrome [1] usually appearing unilaterally and ipsilateral to the cutaneous malformation [2]. A diffuse choroidal hemangioma is a risk factor for secondary exudative or hemorrhagic retinal detachment during or after intraocular surgery [3].

There is no definitive treatment regimen for exudative retinal detachment in patients with choroidal hemangiomas. Treatment options include radiotherapy [4], photodynamic therapy [5], oral propranolol [6], pegaptinib [7], and bevacizumab [8],[9].

## Case presentation

A 26-year-old white male developed a decrease of vision with associated photopsias in his left eye (OS). The symptoms developed minutes after he took an over-the-counter arginine supplement advertised for erectile dysfunction. The patient’s medical history was significant for Sturge-Weber Syndrome with a left nevus flammeus affecting the ophthalmic and maxillary division of the trigeminal nerve. Other than the cutaneous symptoms, the patient had no hypertension or blood dyscrasias. Ophthalmologic history was remarkable for a diffuse choroidal hemangioma in his left eye with associated mild amblyopia. The patient had no surgical history. At initial evaluation, the patient was using one drop of nepafenac 0.1% to the left eye 3 times a day, which was prescribed by his primary ophthalmologist for the exudative retinal detachment for 5 days. The patient was not taking any other prescriptions or supplements. The patient also denies any trauma or strenuous activity preceding the decrease in vision. The patient’s last ophthalmologic exam was in the year prior to the inciting event with no sign of an exudative retinal detachment as per the patient’s primary ophthalmologist.

**Figure 1:**
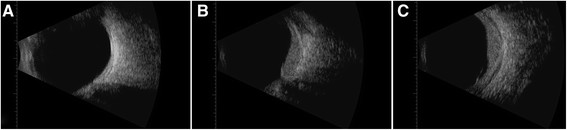
**B-scan of both eyes. (A)** Normal eye, OD, **(B)** longitudinal, and **(C)** transverse of OS showing diffuse choroidal thickening, and internal reflectivity representing a diffuse choroidal hemangioma with peripheral retinal detachment.

**Figure 2:**
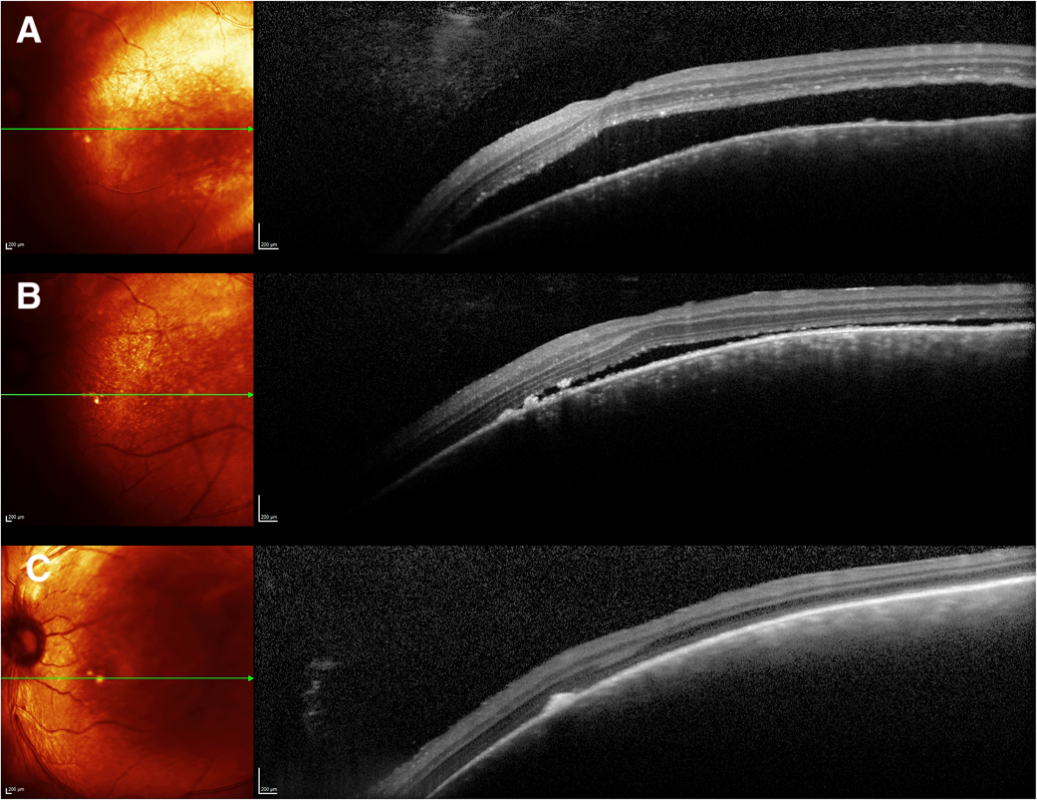
**SD**-**OCT with corresponding multicolor images. (A)** SD-OCT with corresponding multicolor image of subretinal fluid associated with hemangioma before treatment. **(B)** SD-OCT with corresponding multicolor image of subretinal fluid associated with hemangioma after first intravitreal Avastin treatment, **(C)** SD-OCT with corresponding multicolor image of subretinal fluid associated with hemangioma after four intravitreal Avastin treatments.

**Figure 3:**
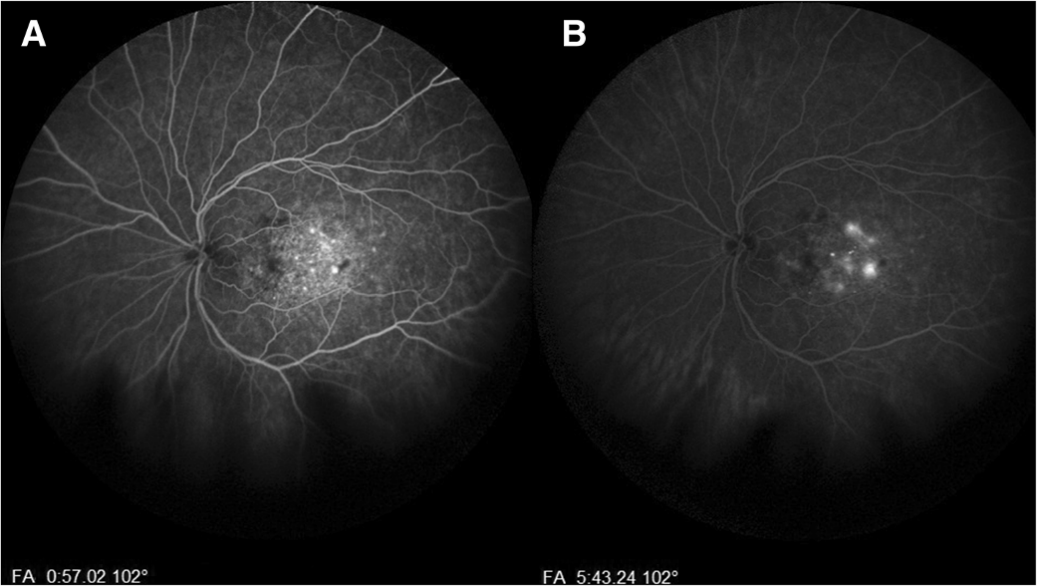
**Fluorescein angiography of the left eye. (A)** Marked central hyperfluorescence in the venous phase, and **(B)** late phase showing increased hyperfluorescence indication leakage.

A complete ophthalmologic exam was performed. Best-corrected visual acuity was 20/20 right eye (OD) and 20/40 OS. Intraocular pressures were 13 and 15 mmHg, respectively. Pupils were equally round and reactive. Visual fields, anterior segment, and extraocular motility were unremarkable. External examination was positive for a left facial nevus flammeus. Indirect ophthalmoscopy of the OD fundus was unremarkable. The OS was remarkable for a diffuse, shallow choroidal mass. The cup to disc ratio was 0.3 OD and 0.45 OS. Ocular ultrasonography revealed a slightly irregular, diffuse, dome-shaped mass 3.5 mm thick OS with internal reflectivity, consistent with a diffuse choroidal hemangioma; additionally, a thin, highly reflective membrane suggestive of an overlying exudative retinal detachment was present (Figure 1). Spectral-domain optical coherence tomography (SD-OCT) confirmed the presence of subretinal fluid (Figure 2A). Fluorescein angiography (Figure 3) showed early hyperfluorescence as well as late phase leakge, which is consistent with an exudative retinal detachment. The patient was treated with intravitreal bevacizumab, 1.25 mg in 0.05 mL; six weeks later, SD-OCT showed a marked decrease of the subretinal fluid (Figure 2B). Over the next few months, the patient was treated with further intravitreal Avastin injections with decreasing exudate at each visit.

At six months follow-up, the patient had received 4 intravitreal doses of bevacizumab due to persistent subretinal fluid. Best-corrected visual acuity at that evaluation was 20/40 OS. Resolution of the exudative retinal detachment can be seen in Figure 2C. The patient was seen again at 8 months with continued resolution of the exudative retinal detachment.

## Conclusion

Diffuse choroidal hemangiomas have been reported in up to 71% of patients with Sturge-Weber syndrome [9]. Patients with Sturge-Weber syndrome that have port-wine stains of the eyelids (upper more than lower), bilateral distribution of the port wine stain, or unilateral port-wine stain involving all three distributions of the trigeminal nerve have a greater likelihood of having ocular and neural complications [10]. Sturge-Weber Syndrome can be associated with glaucomatous changes due to increased episcleral outflow obstruction [11]-[13]. Choroidal hemangiomas are commonly associated to visual acuity loss due to refractive amblyopia, glaucoma, macular edema, and exudative retinal detachments [14].

Diffuse choroidal hemangioma remains a diagnostic challenge. The choroidal hemangiomas in Sturge-Weber may sometimes be overlooked because of their diffuse nature and because the hemangioma may blend imperceptibly with the adjacent choroid. Indirect ophthalmoscopy shows increased tortuosity of the retinal vasculature as well as choroidal thickening. Lesions typically present as a diffusely, deep red, “tomato ketchup” fundus. The actual lesion will have ill-defined borders and may involve the entire fundus [2]. Ancillary testing is important in the diagnosis of diffuse choroidal hemangioma. Fluorescein angiography typically shows an early diffuse speckled hyperfluorescent choroidal flush [2]. Echography is associated with a hyperechoic lesion with high internal reflectivity and diffuse choroidal thickening [15]. Optical coherence tomography will show enhanced visualization of the choroid.

Patients with diffuse choroidal hemangiomas are at higher risk for the development of spontaneous or drug-induced exudative retinal detachments. Vascular alterations in retina and choroid may play a key role in the pathogenesis of the detachment [16]. Recent reports have documented successful treatment of exudative retinal detachments with intravitreal injection of human monoclonal antibodies against vascular endothelial growth factor (anti-VEGF) both as primary and secondary treatments [7],[9],[17]. This therapy is used to inhibit the formation of aberrant blood vessels and to decrease vascular permeability.

The treatment options for an exudative retinal detachment secondary to a choroidal hemangioma include photodynamic therapy (PDT), intravitreal injection of a vascular endothelial growth factor inhibitor, like Avastin, or a combination of both therapies. The choice to use Avastin only was due to the fact that it is less invasive and has fewer systemic effects. PDT involves intravenous administration of chemicals, which have multiple side effects including, but not limited to the patient not being allowed in the sun for 72 hours after injection. Avastin is a safe alternative, which has been shown equally effective [17]. In general, we do not use combination therapy unless monotherapy with an intravitreal injection does not show adequate improvement.

To our knowledge, this is the first case of a patient with a diffuse choroidal hemangioma that developed an exudative retinal detachment associated to arginine supplementation. Choroidal hemangiomas have been associated to increased VEGF levels [18]. Arginine is a chemical precursor to nitric oxide, which causes vasodilation. Arginine may increase ocular venous dilation and decrease venous return to the systemic circulation. This can lead to both accumulation of fluid in the subretinal space and increase in intraocular pressure.

Erectile dysfunction medications including phosphodiesterase type 5 inhibitors, such as sildenafil, have been associated with numerous ocular side effects including elevation of intraocular pressure, choroidal thickening, and central serous chorioretinopathy [19]-[22]. The major active ingredient of these drugs is an isomer of Arginine. This causes an increase of systemic nitric oxide, which leads to the erection, as well as the exudative retinal detachment, as we postulate. Arginine supplementation may be associated to similar risks in susceptible patients as it is a precursor to nitric oxide [23]. It is also extremely well absorbed and can be absorbed even more quickly with the addition of other supplements. Without the addition of other supplements, such as glutamine, arginine has 68% oral bioavailability, its half-life is 0.7-1.3 hours, and its peak serum time is about 2 hours [24]. There is very little research in regards to the relationship of arginine/nitric oxide to exudative retinal detachments due to arginine not being a standard medical treatment.

In patients with diffuse choroidal hemangiomas, treatment with bevacizumab may improve resolution of subretinal fluid as demonstrated in Figure 2C. Review of medication history and discontinuation of recently added vasodilators may play a key role in the management of patients with exudative detachments associated to choroidal hemangiomas. Our treatment plan of multiple injections of bevacizumab showed that this class of drugs is useful for treatment of exudative retinal detachment secondary to nitric oxide producing vasodilators. Though this exudative retinal detachment can be considered spontaneous, the fact that it occurred in close proximity to the use of a drug from a class that can cause exudative retinal detachments leads us to conclude that this was not a spontaneous exudative retinal detachment. There is also the possibility of spontaneous resolution of the exudative retinal detachment which could have aided Avastin in its resolution.

## Consent

Written informed consent was obtained from the patient for the publication of this report and any accompanying images.

## Authors’ original submitted files for images

Below are the links to the authors’ original submitted files for images. Authors’ original file for figure 1 Authors’ original file for figure 2 Authors’ original file for figure 3
