# Supplementary figures and images for: Spontaneous exudative retinal detachment in a patient with sturge-weber syndrome after taking arginine, a supplement for erectile dysfunction
Source: Eye Vis (Lond). 2014 Oct 22;1:7. doi: 10.1186/s40662-014-0007-x (PMC4657225; doi:10.1186/s40662-014-0007-x)

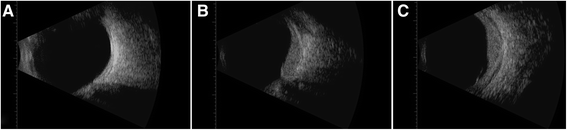

Supplement: Supplementary file 1 — Authors’ original file for figure 1 [file 40662_2014_7_MOESM1_ESM.gif]

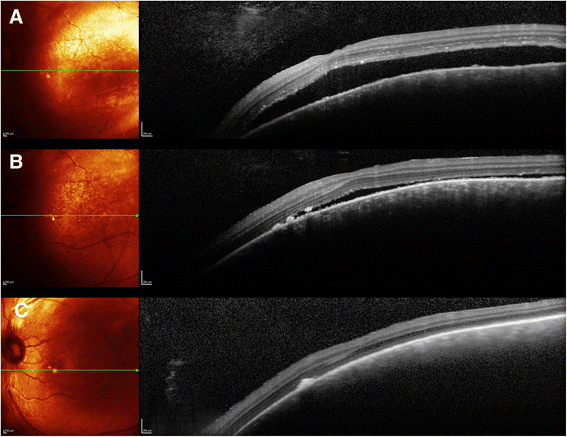

Supplement: Supplementary file 2 — Authors’ original file for figure 2 [file 40662_2014_7_MOESM2_ESM.gif]

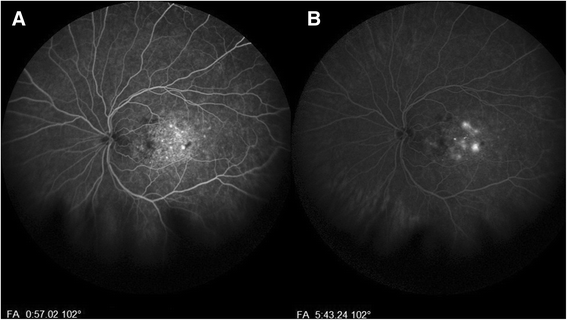

Supplement: Supplementary file 3 — Authors’ original file for figure 3 [file 40662_2014_7_MOESM3_ESM.gif]
